# Additive Manufacturing of Poly(3-hydroxybutyrate-*co*-3-hydroxyvalerate)/Poly(D,L-lactide-*co*-glycolide) Biphasic Scaffolds for Bone Tissue Regeneration

**DOI:** 10.3390/ijms23073895

**Published:** 2022-03-31

**Authors:** Gianni Pecorini, Simona Braccini, Gianluca Parrini, Federica Chiellini, Dario Puppi

**Affiliations:** 1 BIOLab Research Group, Department of Chemistry and Industrial Chemistry, University of Pisa, UdR INSTM Pisa, Via Moruzzi 13, 56124 Pisa, Italy; gianni.pecorini@phd.unipi.it (G.P.); simona.braccini@phd.unipi.it (S.B.); federica.chiellini@unipi.it (F.C.); 2 Fabrica Machinale, Via Giuntini 13, Cascina, 56021 Pisa, Italy; gparrini@smrobotica.it

**Keywords:** polyhydroxyalkanoates, tissue engineering, additive manufacturing, 3D printing, biphasic scaffolds, poly(3-hydroxybutyrate-*co*-3-hydroxyvalerate), polyhydroxyalkanoate blends, computer-aided wet-spinning

## Abstract

Polyhydroxyalkanoates are biopolyesters whose biocompatibility, biodegradability, environmental sustainability, processing versatility, and mechanical properties make them unique scaffolding polymer candidates for tissue engineering. The development of innovative biomaterials suitable for advanced Additive Manufacturing (AM) offers new opportunities for the fabrication of customizable tissue engineering scaffolds. In particular, the blending of polymers represents a useful strategy to develop AM scaffolding materials tailored to bone tissue engineering. In this study, scaffolds from polymeric blends consisting of poly(3-hydroxybutyrate-*co*-3-hydroxyvalerate) (PHBV) and poly(D,L-lactide-*co*-glycolide) (PLGA) were fabricated employing a solution-extrusion AM technique, referred to as Computer-Aided Wet-Spinning (CAWS). The scaffold fibers were constituted by a biphasic system composed of a continuous PHBV matrix and a dispersed PLGA phase which established a microfibrillar morphology. The influence of the blend composition on the scaffold morphological, physicochemical, and biological properties was demonstrated by means of different characterization techniques. In particular, increasing the content of PLGA in the starting solution resulted in an increase in the pore size, the wettability, and the thermal stability of the scaffolds. Overall, in vitro biological experiments indicated the suitability of the scaffolds to support murine preosteoblast cell colonization and differentiation towards an osteoblastic phenotype, highlighting higher proliferation for scaffolds richer in PLGA.

## 1. Introduction

Bone tissue lesions are among the most common injuries of the human body. The increased need for bone replacement has become a key scientific, socioeconomic, and clinical challenge. Tissue engineering (TE)-based strategies are providing reliable therapeutic tools for healing damaged bone tissue [1]. For this reason, the past decades have seen a dramatic increase in research on novel biodegradable materials for bone scaffold development [2]. Together with the increasing environmental awareness and need for sustainable production, this is also encouraging research on biopolymers in the field of bone TE [3].

Polyhydroxyalkanoates (PHAs) are aliphatic polyesters synthesized by many Gram-positive and Gram-negative bacteria under unbalanced growth conditions. PHA monomeric units typically hold as a side-chain R group a saturated alkyl group, varying from methyl to tridecyl [4]. Thanks to properties such as biocompatibility, biodegradability, and better mechanical properties in comparison with other biopolymers, PHAs are unique candidates for advanced research and development approaches in biomedical applications such as TE [5]. Even though poly(3-hydroxybutyrate) (PHB) and poly(3-hydroxybutyrate-*co*-3-hydroxyvalerate) (PHBV) have found application in biomedicine, PHAs are not yet fully utilized in this context, mainly due to their limited processing properties, brittle mechanical behavior [6], and relatively slow degradation [7]. Combining different materials is a useful way to increase their physicochemical and biological properties and optimize them for specific applications [8,9,10]. Blending a PHA with other polymers is a straightforward and versatile strategy to obtain new materials with overall superior properties in comparison to those of the starting macromolecular compounds [6]. For instance, blending PHB or PHBV with other polymers or low molecular weight compounds is reported to influence the crystallinity and mechanical properties of the final material, thus expanding its possible applications [11,12,13,14,15,16]. The intrinsic hydrophobicity of PHB and PHBV is another factor that could restrict their applications in TE, because it negatively affects cell attachment to the scaffold, resulting in an inadequate cell colonization of the porous structure. Blending PHAs with more hydrophilic polymers, such as poly(ethylene oxide)-based copolymers, represents an effective strategy to increase the hydrophilicity of the final material, making it suitable for the development of scaffolds for TE [17]. Hydrolytic ester bond scission mainly contributes to PHA degradation in vitro and in vivo [7]. Moreover, the final degradation products of PHB and PHBV are natural human blood constituents and thus they exert low inflammatory action. A recent article reported the investigation of the in vitro degradation of PHBV porous scaffolds, showing that PHBV molecular weight decreased to 92.6% of its original value after 12 weeks of incubation in a Phosphate Buffer Saline (PBS) at 37 °C [18]. However, the PHBV degradation rate depends on many factors, such as the scaffold structure (e.g., architecture, pore size, porosity); environmental conditions (e.g., incubation medium, site of implantation, temperature, pH value); and material properties (e.g., molecular weight, copolymer composition, crystallinity, hydrophilicity). The blend formation is a valuable way to tune the crystallinity and the hydrophilicity of polymeric materials, and in this way the degradation kinetics of the resulting scaffolds can be optimized and tailored to the regeneration rate of the target tissue.

PHA processability, both as a melt or solution/suspension in a suitable organic solvent, has allowed the investigation and application of a wide range of processing techniques relevant to biomedical research and industrial applications, such as Additive Manufacturing (AM) technologies [19]. AM enables the fabrication of 3D structures with predefined geometry and external size, as well as with a porous architecture characterized by a fully interconnected network of pores with customizable size, shape, and distribution [20]. The control of the scaffold’s architecture represents an effective approach to tailor its properties. A representative example is given by scaffolds with triply periodic minimal surface architecture, whose mechanical properties can be optimized in a very precise way by varying their porosity, wall thickness, and cell size [21]. Different AM techniques have been investigated to fabricate PHA scaffolds for bone tissue engineering. PHB and PHBV have been processed by Selective Laser Sintering (SLS) to fabricate scaffolds loaded with inorganic osteoconductive fillers and osteogenic growth factors [22,23,24,25,26,27,28] and, in some cases, endowed with an anatomical shape [29]. PHB, PHBV, and poly(3-hydroxybutyrate-*co*-3-hydroxyhexanoate) (PHBHHx) have been processed by Fused Deposition Modeling (FDM), often as blends with polymers with better rheological properties or low-molecular weight plasticizers [11,30,31,32,33]. PHBHHx is the only PHA with thermal and rheological properties that make it suitable for direct processing by FDM into 3D structures with controlled architecture [30]. Conversely, PHB and PHBV have been processed by FDM only as blends with other thermoplastic polymers, such as poly(ε-caprolactone) (PCL) and poly(lactide) (PLA). In addition, to the best of the authors’ knowledge, PHBHHx is the only reported PHA that has been successfully processed by AM approaches based on solution/suspension extrusion. In particular, a number of articles have described the employment of Computer-Aided Wet-Spinning (CAWS) to fabricate bone scaffolds made of PHBHHx or PHBHHx/PCL blends [34,35,36,37]. CAWS is an AM technique based on the extrusion and controlled deposition of a polymeric solution or suspension directly into a coagulation bath. The mechanism of fiber formation involves a solvent/nonsolvent exchange that can lead to the separation of the initial polymer solution in a polymer-lean and a polymer-rich phase [38]. After solidification, as a result of the phase separation process, microporous polymeric fibers can be obtained [5,38].

The aim of this work was to investigate for the first time the suitability of CAWS for processing PHBV, possibly blended with poly(D,L-lactide-*co*-glycolide) (PLGA), as a scaffolding material for bone regeneration. PLGA is an aliphatic biodegradable copolyester that has been widely investigated for biomedical applications, receiving FDA approval for drug delivery and other clinical uses, such as absorbable sutures and fixation devices for bone fracture stabilization and craniofacial reconstruction [39,40]. The biodegradation of PLGA is caused by the hydrolytic cleavage of ester bonds, as described by a recent article reporting that the molecular weight of PLGA constituting porous scaffolds decreased by about 89% of its original value after 95 days of incubation in PBS [41]. The CAWS parameters for the fabrication of scaffolds with a predefined shape, size, and porous architecture were optimized. The fabricated scaffolds were characterized by means of scanning electron microscopy (SEM), thermogravimetric analysis (TGA), differential scanning calorimetry (DSC), proton nuclear magnetic resonance (^1^HNMR), contact angle measurements, uniaxial compression tests, and in vitro biological evaluation employing MC3T3-E1 murine preosteoblast cells. 

## 2. Results

### 2.1. Scaffold Fabrication

**Figure 1:**
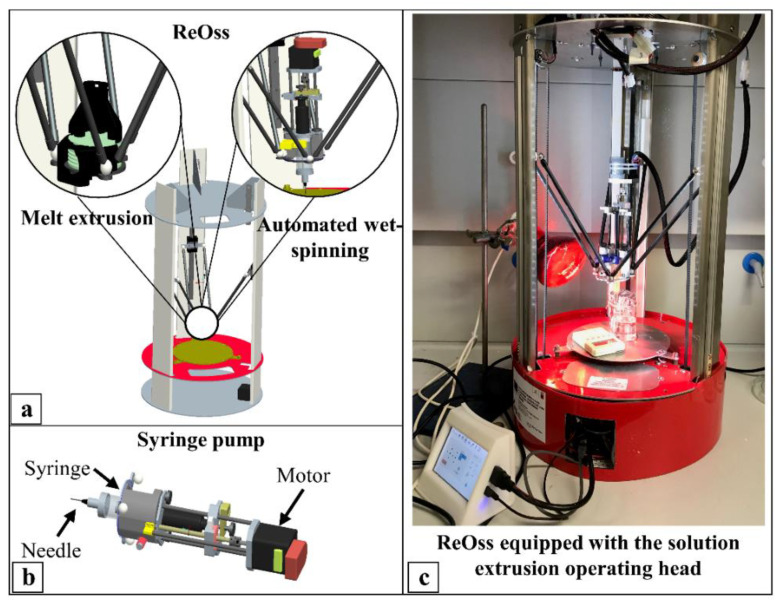
Additive Manufacturing (AM) equipment: (**a**) schematic representation of the AM system and the two switchable operating heads to employ either melt-extrusion or solution-extrusion AM, i.e., Computer-Aided Wet-Spinning (CAWS); (**b**) schematic representation of the CAWS extrusion head employed in this study, showing the syringe equipped with a blunt-tip needle and housed in a feed-controlling pump; (**c**) representative picture of the ReOss apparatus equipped with the syringe pump for CAWS processing (the lamp in the background was employed to control the temperature of the syringe and the coagulation bath).

The design and fabrication of the scaffolds made of PHBV or PHBV/PLGA blends were carried out by CAWS. A novel AM apparatus allowing for the use of both melt- and solution-extrusion AM by easily interchanging the deposition head through magnetic connections was employed for this purpose (Figure 1).

**Figure 2:**
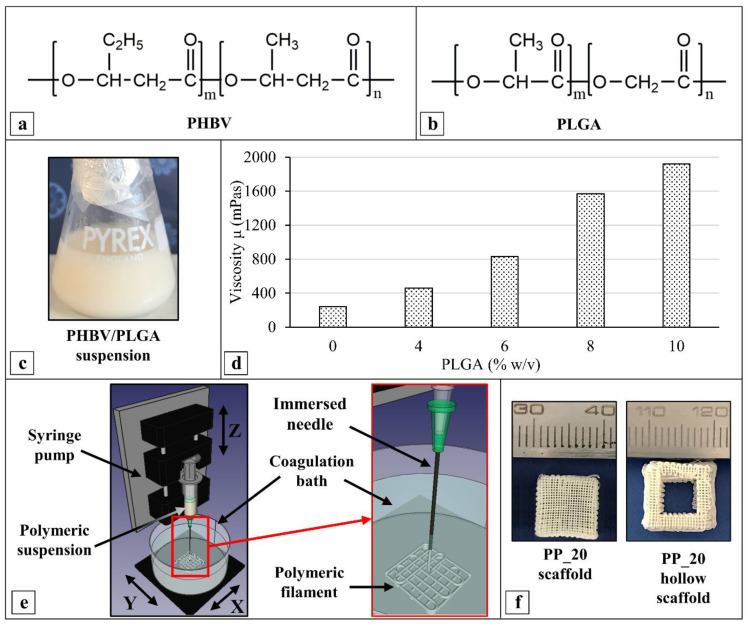
Chemical structure of the two aliphatic polyesters employed in this study, i.e., (**a**) poly(3-hydroxybutyrate-*co*-3-hydroxyvalerate) (PHBV) and (**b**) poly(D,L-lactide-*co*-glycolide) (PLGA); (**c**) representative picture of a PHBV suspension in a PLGA chloroform solution (PHBV/PLGA 80:20 suspension) employed for scaffold fabrication by CAWS; (**d**) viscosity of PHBV/PLGA suspensions as a function of PLGA concentration (only a single measurement was carried for each kind of sample due to the large volume of suspension, i.e., 50 mL, and relevant polymer mass, i.e., 20 g, required for analysis); (**e**) schematics of the CAWS process highlighting details about polymeric mixture extrusion directly into a coagulation bath and the layer-by-layer deposition of the solidifying fiber; (**f**) representative pictures of PP_20 scaffolds with standard shape (**left**) as well as hollow geometry designed and fabricated as a proof of concept of CAWS process versatility (**right**).

A set of polymeric suspensions (Figure 2a–c) with an overall polymer concentration of 20% *w*/*v* and different PHBV/PLGA weight ratios (i.e., 100:0, 90:10, 80:20, 70:30, 60:40, and 50:50) (Table 1) were investigated for scaffold fabrication. The dynamic viscosity (µ) measurements revealed an increase in the suspension’s viscosity from around 240 to 1920 mPas by increasing the PLGA weight percentage from 0 to 50% (Figure 2d). The CAWS approach involved the deposition, in a predefined pattern, of the extruded polymeric suspension into a coagulation bath to make 3D scaffolds by means of a layer-by-layer process (Figure 2e). The solidification of the wet-spun polymeric fiber is governed by a non-solvent-induced phase-separation process, caused by polymer desolvation, as a consequence of solvent–nonsolvent counterdiffusion [42]. Different coagulation media, such as ethanol, water, and a mixture ethanol/chloroform (90:10% *v*/*v*), were investigated. The best outcomes in terms of polymer coagulation to form a continuous PHBV or PHBV/PLGA fiber with a uniform morphology were achieved by employing ethanol as the nonsolvent. Prism-shaped scaffolds with a square base were fabricated by optimizing the most influential manufacturing parameters, i.e., the extrusion flow rate (F), needle translation velocity (V_transl_), and coagulation bath temperature (T_coag_). 

Structure delamination and warpage were often observed in the PP_0 and PP_10 samples during scaffold drying under a fume hood, after sample removal from the coagulation bath as usually carried out in CAWS processes [43]. In the case of the PP_20, PP_30, PP_40, and PP_50 samples, only warping deformation was observed. Different postprocessing treatments were investigated by optimizing the experimental protocols aimed at minimizing the aforementioned macroscopic variations in the scaffolds’ structural and geometrical features. In the case of the PP_0 and PP_10 scaffolds, the optimized postprocessing involved their immersion in ethanol for 6 days before drying under a fume hood for 48 h, while in the case of the other scaffolds, pressure was applied on the top surface of the scaffolds during drying under a fume hood for 48 h. After postprocessing treatment, all scaffolds were vacuum dried for 6 h at room temperature. All postprocessing treatments are schematically represented in Figure S1.

PP_20 scaffolds with a customized hollowed geometry were also fabricated by employing the relevant optimized manufacturing parameters, to demonstrate the versatility of the developed CAWS process (Figure 2f). Overall, the developed scaffolds were robust, being able to undergo postprocessing treatments without any damage and withstand routine handling forces.

### 2.2. Morphological Characterization

**Figure 3:**
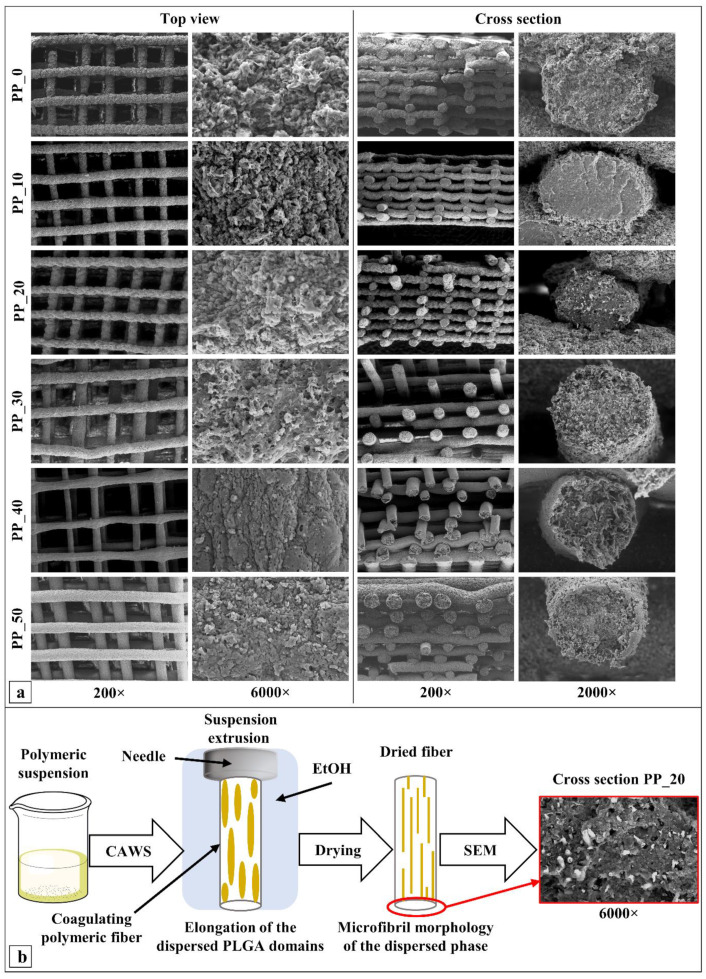
(**a**) Representative top-view (**left**) and cross-section (**right**) SEM micrographs of the fabricated scaffolds with different compositions, taken at different magnifications to highlight the 3D layered porous structure of scaffolds (200×), fiber external surface roughness (6000×), and microporous/microfibrillated morphology of the fiber transversal cross-section (2000×); (**b**) schematic representation of the proposed mechanism for two-phase polymeric matrix formation showing the deformation of the dispersed PLGA solution phase during extrusion, leading to the formation of longitudinally oriented microfibrils dispersed in a continuous polymeric phase, as observed in the included SEM micrograph (6000×).

The morphology of the developed scaffolds was investigated by means of SEM analysis of a sample’s top view and cross-section. The analysis highlighted that the fabricated scaffolds were composed of a 3D layered structure of aligned fibers forming a fully interconnected network of macropores, as shown in Figure 3a, whose average size was between 227 and 360 µm (Table 2). Scaffold PP_40 showed the smallest fiber diameter and largest pore size by a significant margin. The porosity of the fabricated 3D structures fell in the range between 68 and 75%. 

The analysis of the high-magnification micrographs of the scaffolds’ cross-sections revealed that the fibers were composed of two different polymer phases with different chromatic appearances, one being a continuous matrix in which the other was dispersed, in some cases assuming microfibrillar morphology. Microfibrils were randomly distributed and showed a certain degree of alignment along the longitudinal direction of the scaffolds’ fibers. 

### 2.3. Acetone Leaching and ^1^HNMR Analysis

In order to verify the hypothesis that the continuous phase of the polymeric fibers was constituted by PHBV and the dispersed phase by PLGA, a representative PP_20 scaffold was immersed in acetone to selectively extract PLGA.

**Figure 4:**
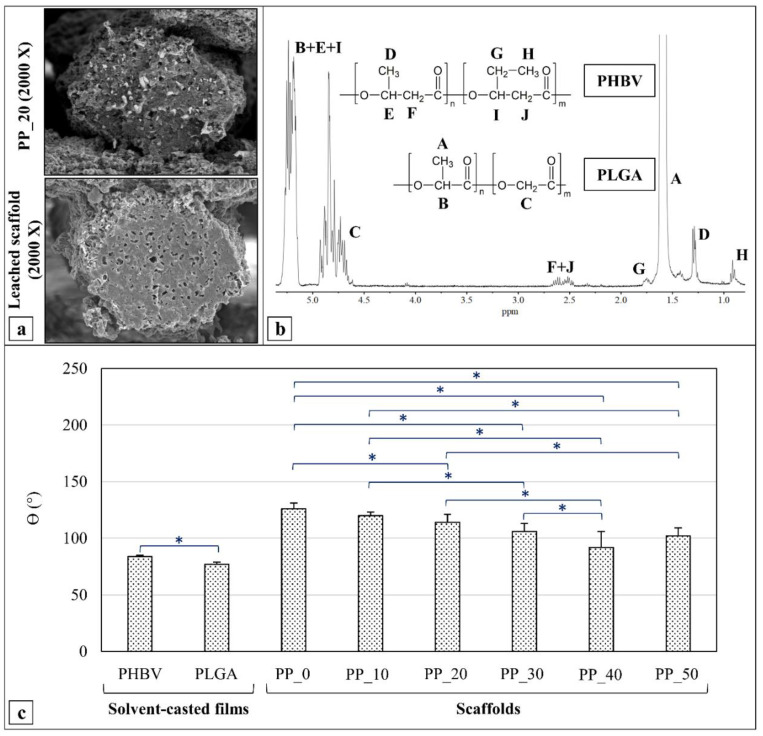
(**a**) SEM micrographs of the cross-section of a single PP_20 scaffold fiber before (top) and after (down) acetone leaching, demonstrating the presence of microholes in place of microfibrils, as a consequence of the solvent selective extraction process; (**b**) ^1^HNMR spectrum of the extracted phase, whose analysis corroborated the hypothesis that the leached-out microfibrillar phase was mainly composed of PLGA; (**c**) contact angle values (θ) obtained for PHBV films, PLGA films, and PHBV/PLGA scaffolds, showing the higher hydrophobicity of scaffolds due to macroporosity and surface roughness (measurements carried out on ten samples for each kind of scaffold). * Values statistically different (*p* < 0.05).

SEM analysis (Figure 4a) was carried out on the leached scaffold, while the extract was analyzed by ^1^HNMR. The ^1^HNMR spectrum of the extract (Figure 4b) revealed signals assigned to the two polymers that constituted the blend [44,45]. The multiplets at 5.2 ppm and 4.8 ppm and the singlet at 1.6 ppm were assigned, respectively, to the protons of the methine group, methylene group, and methyl group of PLGA; the multiplets at 2.6 ppm, 1.7 ppm, 1.3 ppm, and 0.9 ppm were assigned, respectively, to the protons of the methylene groups of the PHBV main chain, the side methylene group of 3-hydroxyvalerate, the methyl groups of 3-hydroxybutyrate, and the methyl groups of 3-hydroxyvalerate monomeric units. The signals corresponding to the protons of the methine groups of PHBV were covered by that of PLGA at 5.2 ppm. The fraction of PLGA in the extracted phase was estimated from the relative integrated methylene signal (4.8 ppm and 2.6 ppm) and resulted in a figure of 99.75 mol.%.

### 2.4. Contact Angle Measurements

The water contact angle (ϴ) of the scaffolds made of PHBV (PP_0) or PHBV/PLGA blends (PP_10, PP_20, PP_30, PP_40, and PP_50) was measured in comparison to that of the solvent-casted films made of the two starting polymers (PHBV and PLGA) (Figure 4c). The ϴ measured for the PHBV film and PLGA film were 84° and 77°, respectively, indicating that PLGA is significantly more hydrophilic than PHBV. 

Measurements carried out on the scaffolds showed an increase in θ values in comparison to those measured on the films; for instance, the PP_0 scaffold exhibited a value of 126°. The statistical analysis revealed a significant decrease in the ϴ as the scaffold’s PLGA concentration increased up to 40 wt%.

### 2.5. Thermal Characterization

**Figure 5:**
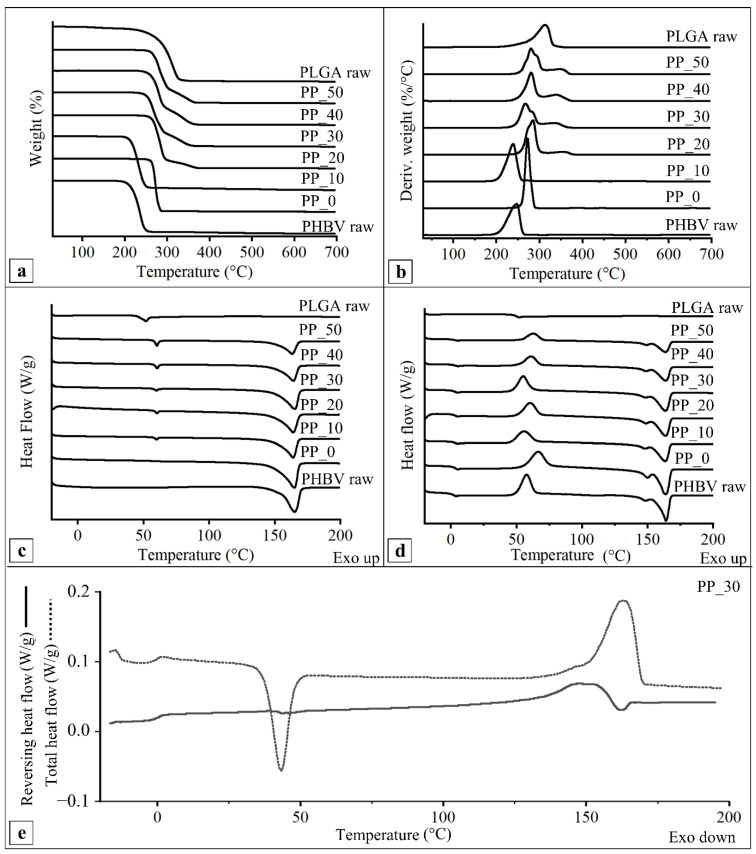
Representative thermograms of raw polymers (raw PHBV and raw PLGA) and scaffolds with different PHBV/PLGA weight ratios (PP_0, PP_10, PP_20, PP_30, PP_40, and PP_50) obtained by Thermogravimetric Analysis (TGA), reported as (**a**) weight loss vs. temperature and (**b**) derivative of weight vs. temperature curves, and by Differential Scanning Calorimetry (DSC), reported as (**c**) first heating scan and (**d**) second heating scan curves; (**e**) representative thermogram obtained by means of modulated DSC analysis and relevant to the second heating scan of the PP_30 scaffold.

The thermal properties of the developed scaffolds were investigated in comparison to those of the raw polymers and their physical mixture. TGA analysis showed that the raw polymer thermograms (Figure 5a,b) were characterized by a single degradation step. Indeed, the derivative curves of the raw polymers showed a single peak centered at around 240 °C (T_max1_) or 310 °C (T_max2_) for PHBV and PLGA, respectively (Table 3). The TGA curves of the PP_0 and PP_10 scaffolds were characterized by one thermal degradation event; specifically, the derivative curves showed a T_max1_ of about 270 °C and 240 °C, respectively (Table 3). The difference in T_max1_ between raw PHBV and the PP_0 scaffold could be due to polymeric material purification during the coagulation process [46]. However, no significant difference in T_max1_ between the PP_10 scaffold and raw PHBV was detected. The TGA curves of the PP_20, PP_30, PP_40, and PP_50 scaffolds showed two distinct weight-loss events, and the derivative curves were characterized by two peaks associated with the thermal degradation of the two polymers making up the blend (T_max1_ and T_max2_) that were significantly higher than those of the raw polymers (Table 3).

The DSC thermograms of the first heating scan (Figure 5c) showed the glass transition temperature (T_g_), melting temperature (T_m_), and melting enthalpy (ΔH_m_) of the raw polymers and the manufactured scaffolds. In particular, the raw PHBV thermogram presented an endothermic peak centered at 164 °C, which was associated with the melting of the crystalline domains, while the raw PLGA thermogram showed a glass transition centered at about 50 °C. The DSC thermograms of the PP_0 scaffold showed a melting peak at 165 °C, while those of the PHBV/PLGA scaffolds showed both a glass transition at about 50 °C and a melting peak at 163–165 °C. 

The DSC thermograms of the second heating scan (Figure 5d) of the raw PLGA showed a glass transition at around 50 °C, while those of the raw PHBV showed a glass transition at about 2.5 °C; an exothermic peak at around 60 °C, characteristic of the cold crystallization of the polymer; and two endothermic peaks, the larger of which was centered at around 160 °C (Table 4), relative to the melting of the polymer’s crystalline domains. 

The DSC second heating thermograms of the fabricated PHBV/PLGA scaffolds presented a T_g_ in the range of 3.1–3.5 °C, as well as a cold crystallization temperature (T_cc_) between 55 and 60 °C and a T_m_ at about 165 °C (Table 4), which were assigned to the first order thermal transitions of the PHBV fraction that constituted the blend. It was not possible to detect the PLGA glass transition, because it fell in the same temperature range as the PHBV cold crystallization exothermic peak. 

The total and reversing heat flow versus the temperature of the second heating scan of the modulated DSC (MDSC) analysis carried out on a representative scaffold (PP_30) are reported in Figure 5e. The total heat flow curve showed the same thermal transitions as the standard DSC, i.e., the PHBV glass transition, cold crystallization, and melting. The reversing heat flow curve highlighted the presence of the glass transition of PHBV at around 0 °C, that of PLGA at around 50 °C, and an endothermic peak at around 149 °C, relative to the PHBV reversing melting. The presence of two distinct glass transitions confirmed the immiscibility of the two polymers constituting the blend.

### 2.6. Mechanical Characterization

**Figure 6:**
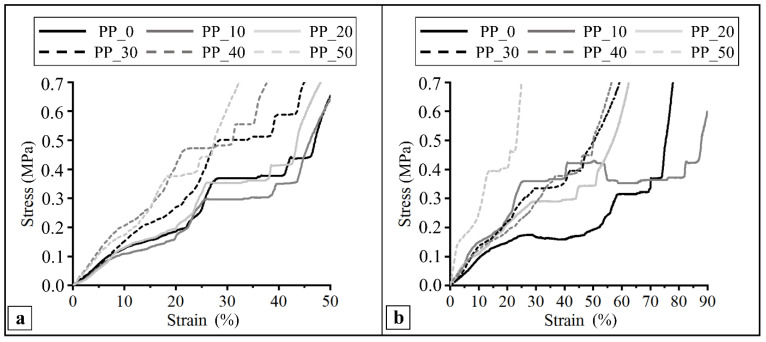
Representative compression stress–strain curves of scaffolds with different PHBV/PLGA weight ratios (PP_0, PP_10, PP_20, PP_30, PP_40, and PP_50) tested (**a**) as dried samples or (**b**) after incubation in PBS 1× at 37 °C, showing the effect of blend composition and testing conditions on mechanical response.

The compressive mechanical properties of the developed scaffolds were analyzed at a constant strain rate by means of a uniaxial testing machine. The test was carried out on dry samples (Figure 6a) as well as after sample incubation in PBS 0.01 M at 37 °C (Figure 6b). In general, the compressive stress–strain curves were characterized by two initial linear regions followed by a plateau and a subsequent region of increasing stress. As reported in Table 5, the PBS treatment did not have a significant effect on the shape of the curves and the resulting mechanical parameters, with the only exception being the PP_10 scaffold, which showed a significantly higher compression modulus. The tested scaffolds showed a compressive modulus (E) in the range of 1–3.7 MPa, a stress at 50% strain (σ_50_) in the range of 0.4–2.2 MPa, and a linear elastic recovery of height (H_Rec_) in the range of 28–51%.

### 2.7. Biological Characterization

**Figure 7:**
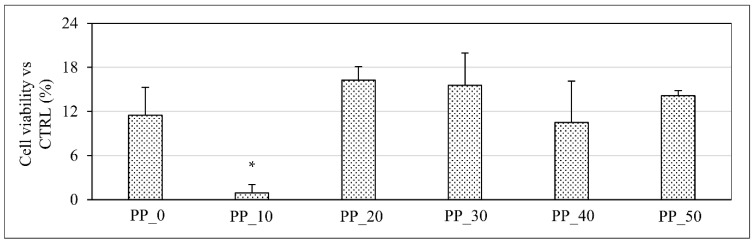
MC3T3-E1 cell viability on scaffolds with different PHBV/PLGA weight ratios (PP_0, PP_10, PP_20, PP_30, PP_40, PP_50) at 2 h of cell culture to quantitatively evaluate the effect of blend composition on cell colonization of scaffolds a short time after seeding (four samples for each kind of scaffold were analyzed). * Value statistically different in comparison to the others (*p* < 0.05).

**Figure 8:**
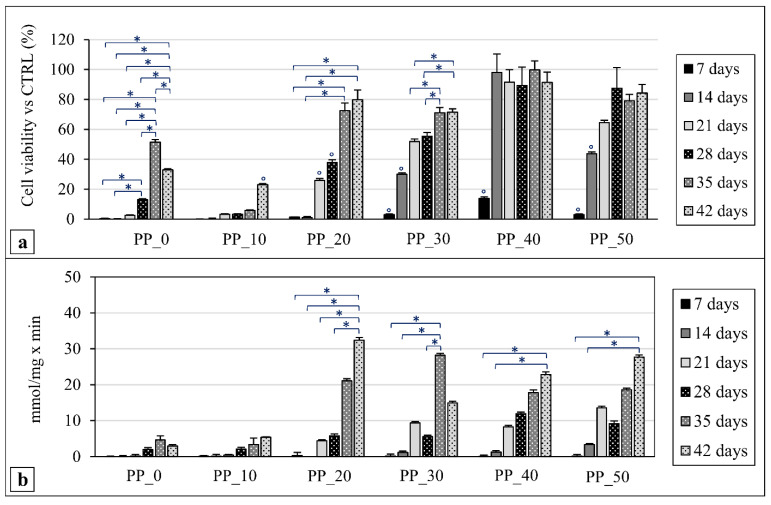
(**a**) MC3T3-E1 cell viability on PHBV/PLGA scaffolds at different time points of cell culture to evaluate the effect of blend composition on cell proliferation over time; (**b**) ALP synthesis as an indicator of MC3T3-E1 cell differentiation towards an osteoblastic phenotype on PHBV/PLGA scaffolds at different time points (three samples for each kind of scaffold were analyzed). The statistical analysis showed in the figures was obtained by comparing the values of scaffolds composed of the same blend at different durations of culture. * Values statistically different (*p* < 0.05). ° Data marked with this symbol are statistically different from all the other values obtained for the same blend composition at different experimental points (*p* < 0.05).

The MC3T3-E1 preosteoblast cell line proliferation and differentiation over time of the culture were assessed for the different kinds of scaffold developed by means of the WST-1 tetrazolium salt assay and alkaline phosphatase (ALP) activity measurements, respectively. The statistical significance of the observed differences was evaluated by a two-way ANOVA test followed by two Tukey post hoc tests. The results achieved are graphically represented in Figure 7 and Figure 8.

Cell viability was first assessed at 2 h of scaffold cell culture (Figure 7) in order to evaluate the different ability of the tested scaffolds to support cell colonization a short time after seeding. No significant differences at this time point were observed among the different scaffolds, except for the PP_10 sample, which showed a value significantly lower in comparison with those of the other samples (Figure 7).

Cell viability for the different kinds of scaffold was also quantified at day 7, 14, 21, 28, 35, and 42 (Figure 8a). A significant increase in cell viability between day 7 and the subsequent time points was found in all cases. However, differences in cell proliferation trends over time were evident by comparing the data from different samples. For instance, while the PP_20 scaffolds showed significantly increasing cell viability values over the experimental time, the PP_40 scaffolds showed only a marked significant increase in cell viability from day 7 to day 14. The statistical analysis of the cell viability differences at each experimental point among the samples with different compositions (not graphically represented in Figure 8a) highlighted that, in general, the increase in PLGA percentage resulted in significantly higher values. As an example, the PP_40 scaffolds after 14 days of culture showed a cell viability value significantly higher than those of the other scaffolds at the same experimental point.

The results from the ALP activity measurements at day 7, 14, 21, 28, 35, and 42 of cell culture are shown in Figure 8b. ALPs are a group of enzymes involved in the cleavage of phosphoric acid monoesters. They are considered an early marker of osteogenesis and are measured to assess the MC3T3-E1 preosteoblast cell differentiation towards the osteoblast phenotype [47]. Cells cultured on the developed scaffolds produced low levels of ALP during the first two endpoints of the analysis. In general, a significant increase in ALP values was observed over time for the PP_20, PP_30, PP_40, and PP_50 samples. The statistical analysis of the differences at each experimental point among the samples with different compositions (not graphically represented in Figure 8b) highlighted also that, after 35 days of culture, the PP_30 scaffolds resulted in a significantly higher ALP value than those of the PP_0 and PP_10 scaffolds, while, after 42 days of culture, the PP_20 and PP_50 scaffolds showed significantly higher ALP values in comparison with those of the PP_0 and PP_10 scaffolds.

**Figure 9:**
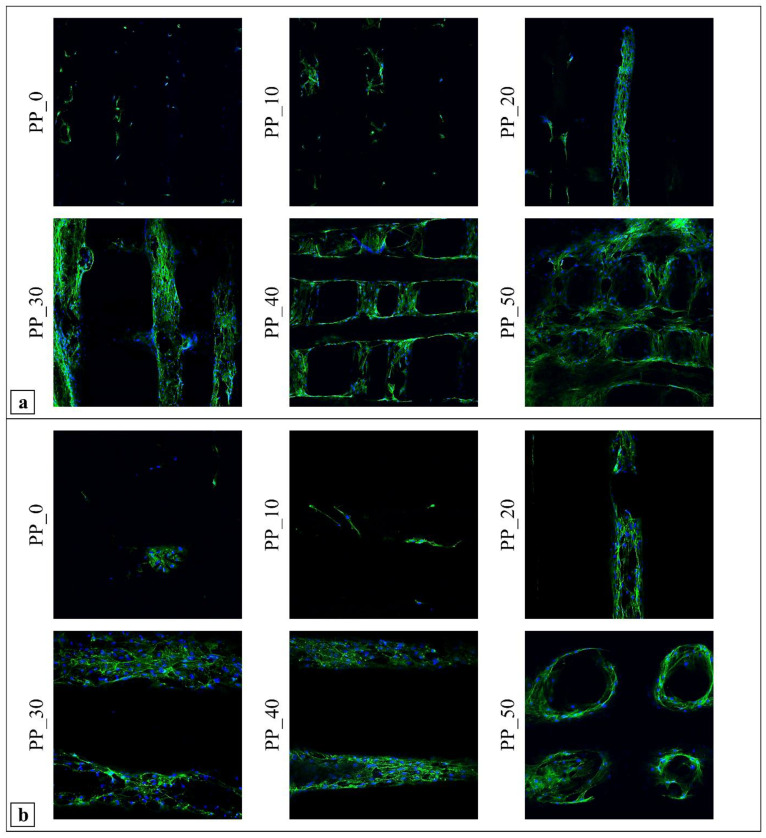
CLSM micrographs showing MC3T3-E1 cells after 28 days of culture on scaffolds with varied PHBV/PLGA weight ratios (PP_0, PP_10, PP_20, PP_30, PP_40, PP_50) taken at different magnifications, (**a**) 10×, (**b**) 20×, highlighting differences in cell colonization by changing the blend composition (cytoskeleton stained with Alexa Fluor 488 phalloidin demonstrating green fluorescence; nuclei stained with blue-emitting fluorescent DAPI).

CLSM was employed to observe the MC3T3-E1 cell morphology and distribution on the investigated scaffolds at day 28 of cell culture by the fluorescent staining of the cytoskeleton (Alexa Fluor 488 phalloidin) and nuclei (DAPI). Micrographs at 10× magnification (Figure 9a) showed higher cell colonization on the PP_20, PP_30, PP_40, and PP_50 scaffolds than on the PP_0 and PP_10 scaffolds. Micrographs taken at 20× magnification (Figure 9b) evidenced that the organization of F-actin was comparable to that typical of the early stages of cellular adaptation to the material, exhibiting great stress fibers stretched along the cytoplasm and confirming good adhesion to the polymer substrates for all analyzed samples [35,48]. The analysis also highlighted the partial cell colonization of the pores of the PP_50 scaffolds as a consequence of cellular bridging between crossing polymeric fibers.

## 3. Discussion

A novel AM apparatus was employed for the first time in this study to design and fabricate PHBV-based scaffolds by means of the CAWS approach. The design of this prototype was tailored to meet the experimental research requirements thanks to the easy interchange between a CAWS and an FDM extrusion head, the possibility of applying an electric field for electrospinning investigations, and the remote control of the fabrication process. The CAWS technique was first described in 2012 [43] as an AM approach relying on a non-solvent-induced phase-inversion process for polymer solidification through the extrusion of an organic solvent solution directly into a coagulation bath. One of the novelties introduced with CAWS was the possibility of endowing scaffolds made of a synthetic or natural aliphatic polyester with a diffuse microporosity in the polymer matrix [35].

Various extrusion-based AM techniques involving either melt or solution/suspension processing have been described in the literature for the fabrication of various scaffold architectures tailored to different tissue engineering purposes. For instance, Freeform Reversible Embedding of Suspended Hydrogel (FRESH) printing involves the extrusion of hydrogels or cell-laden bioinks within a support bath composed of a material that can directly maintain the position of printed structures as they are extruded and cured, while still allowing for the movement of the extruder needle [49]; after printing, the support bath is liquefied to obtain the final manufact [49]. In comparison to standard melt-extrusion AM techniques (e.g., FDM) CAWS can allow a higher fabrication resolution with a resulting fiber diameter as low as 40 μm [34], as well as avoiding the risk of thermal degradation, but with the drawback of potential residual organic solvents that can be harmful to cells. In addition, the dimension and concentration of the micropores obtained by phase-inversion during CAWS processing can be tuned in a certain range by acting on the fabrication and postprocessing parameters. This can allow the tuning of the scaffold properties related to the polymer matrix porosity, such as the biodegradation rate, cell–material interaction, and drug-release kinetics. On the other hand, the generation of microporosity typically leads to a lower material stiffness, due to the increased specific void volume [19]. Melt-electrospinning writing, another widely used melt-extrusion AM approach, enables the obtainment of submicrometric fibers; however, it results in a small scaffold thickness, which is different from what is achievable with FDM or CAWS in terms of clinically relevant sizes along the three dimensions [19,50].

The developed fabrication protocol relies on mixing PHBV or a PHBV/PLGA blend with chloroform to obtain a polymeric suspension processable by CAWS. Chloroform was selected as solvent thanks to its high solvation power both for PLGA and PHBV. Indeed, it is reported that chlorinated organic molecules, such as chloroform and dichloromethane, are good solvents for PHAs [51]. This behavior is the result of a polar interaction between the chlorine atom of the solvent and the carbon that holds the carbonyl function of the polymer. Moreover, the hydrogen atom of the halogenated solvent is linked to the carbonyl function of the polymer. However, the PHA investigated in this study was only partially dissolved in chloroform, unlike the PLGA, which was completely dissolved. This finding can be explained with the relatively high concentration of PHBV and its high crystallinity degree.

Optimized PHBV/PLGA suspensions with different compositions were successfully processed by means of a novel AM apparatus, allowing the design and fabrication of customized polymeric scaffolds through either melt- or solution-extrusion. The increase in the suspension’s viscosity due to an increase in the PLGA percentage in the starting suspension influenced the optimized CAWS manufacturing parameters. In particular, the PLGA-containing suspensions required an increment in the solution feed rate (F); a PLGA percentage increase required an increase in the T_coag_; and, in the case of the highest PLGA percentage (PP_50); the V_transl_ was decreased by half (Table 1). The postprocessing treatments were optimized to avoid the delamination and the warpage of the fabricated scaffolds. These effects could be due to residual coagulation stresses in the layered structure as a consequence of polymer shrinking during the phase-inversion and drying steps [46].

Morphological characterization through SEM analysis confirmed that the fabricated scaffolds had a fully interconnected network of pores and that the blend composition affected the fiber morphology. Indeed, the fiber surface became rougher for the blends richer in PHBV. Many articles have demonstrated that pore dimensions larger than 150 µm and a porosity greater than 50% provide enough space for bone tissue ingrowth and cell colonization [52,53,54]. As a consequence, the porous architecture features of the scaffolds developed in this work are suitable for bone tissue regeneration. The fiber diameter of the PP_40 scaffolds, which was the smallest by a significant margin, could be related to the high solution viscosity, a parameter affecting the coagulation kinetics and stretching forces acting on the solidifying filament. A further increase in the solution viscosity for the PP_50 scaffolds did not lead to a lower fiber diameter, due to the different fabrication parameters employed (i.e., V_transl_ and T_coag_). The comparative analysis of the SEM micrographs (Figure 3a) of the scaffolds’ cross-sections before and after leaching showed that microholes were detectable in place of microfibrils as a consequence of acetone incubation, confirming the complete dissolution of the dispersed phase. The ^1^HNMR analysis corroborated the initial hypothesis that the dispersed fibrils were made mainly of PLGA. The formation of this fibrillar morphology was likely a consequence of the shearing forces acting on the viscous polymeric mixture due to friction with needle’s inner wall (Figure 3b).

In addition to the morphology, surface properties such as hydrophilicity significantly affect scaffolds’ performance and cytocompatibility [55,56]. The higher contact angle (θ) values of the scaffolds in comparison to the polymeric films is explained by the presence of a multiscale surface morphology (i.e., macroporosity and microscale roughness), according to the Cassie–Baxter and Wenzel equations [57]. The trend observed with the scaffolds’ θ values could be attributed to different causes, such as the higher hydrophilicity of PLGA and the variation in pore size. Indeed, it was found that increasing the scaffolds’ pore size caused an increase in the measured θ values until they reached a maximum and then decreased, as a consequence of the increased water/air interface area [58]. Another reason could be the different fiber surface morphology. Indeed, according to Wenzel equation, the rougher the surface, the higher the value of θ will be [57].

TGA characterization allowed the analysis of the two degradation steps related to the polymers constituting the scaffolds. It is known that the thermal degradation of PHBV occurs almost exclusively by a nonradical random chain scission mechanism, including the formation of a six-membered ring transition state obtained by the interaction between the oxygen atom of the carboxylic group and the hydrogen atom bonded to the α-carbon (in respect to the carbonyl group) [59,60]. PLGA thermal degradation products are similar to those of neat poly(lactide) (PLA) and poly(glycolide) (PGA). These polyesters exhibit a degradation mechanism that can involve a random chain scission at the beginning of the decomposition followed by a specific chain scission at the end of the process [61,62]. The analysis revealed an increase in the PP_0 T_max1_ value in comparison to that of raw PHBV. This could be due to the polymer chain orientation along the longitudinal axis of the scaffold’s fibers, as a result of frictional shear stresses acting on the suspension at the needle inner wall during extrusion. Indeed, it was reported that the polymer chain orientation can increase the intermolecular interactions, with the overall result of enhanced polymer thermal stability [63]. A decrease in the T_max1_ of the PP_10 scaffolds in comparison to that of the PP_0 scaffolds was also detected, likely as a consequence of a decrease in the material’s crystallinity, as shown by DSC analysis. However, an increase in the PLGA content in the blend determined an increase in the T_max1_. A possible explanation for this might be that the reduction in the scaffold’s crystallinity is compensated for by the formation of copolymers between PHBV and PLGA through a transesterification reaction caused by a temperature increase during the TGA analysis. Transesterification is a nucleophilic substitution reaction that can occur between ester groups, resulting in their recombination and the formation of new ester bonds [64]. This process may have led to the formation of copolymers with a T_max_ intermediate between those of PHBV and PLGA.

The DSC analysis of PHBV highlighted a single broad melting peak during the first heating scan and a two-step melting behavior during the second heating scan, as already reported for this copolymer [65,66,67,68] and others belonging to the PHA family, such as PHBHHx [34]. Some authors have hypothesized that the first melting peak in the second scan corresponds to the melting of more defective or smaller crystals, which are able to recrystallize during the heating run, thus forming more perfect crystals that subsequently melt at higher temperatures (second melting peak) [67,68]. The progressive reduction in ΔH_m_ observed in the two heating scans of the scaffolds with different blend compositions can be associated with the decrease in the PHBV concentration, which is highly crystalline, and the increase in the PLGA concentration, which is an amorphous polymer. The polymer crystallinity influences different properties of scaffolds made of aliphatic polyesters, such as their mechanical behavior and their biodegradation rate [68,69]. Considering the glass transition showed by the DSC analysis, the increment in the T_g_ values of the scaffolds in comparison with that of raw PHBV could be due to polymer purification during coagulation in ethanol, which eliminated the polymer impurities acting as plasticizers, corroborating the results from the TGA analysis. Thermal events relevant to the evaporation of residual chloroform (normal boiling point of 61 °C) or ethanol (boiling point of 78 °C) were not observed in the TGA and DSC thermograms, demonstrating the complete removal of solvents by means of the applied postprocessing treatments.

As hypothesized in previous articles on biodegradable polymeric scaffolds obtained by CAWS [34,70], the initial linear regions of the stress–strain compressive graphs could be attributed to the response of the fiber–fiber contact areas to the applied deformation, the plateau to the subsequent collapse of the porous network, and the final continuous stress increase to the densification of the polymeric structure that behaves like a dense matrix. For both test conditions (i.e., dried or after incubation in PBS), the PP_50 scaffold showed the highest compressive modulus, highlighting the influence of the differences in macrostructural features, as observed by SEM analysis, on the resulting mechanical properties. The values of the compression modulus reported in the literature are in the range of 0.1–2 GPa for cancellous bone and 15–50 GPa for cortical bone [71]. The mechanical properties of the fabricated scaffolds are significantly lower than those of human bone tissues; however, it should be considered that bone tissue regeneration starts with the secretion of an unmineralized organic bone matrix, called osteoid, containing a high amount of collagen [35,72]. The Young modulus of the osteoid is 20–50 kPa [73] which is even lower than that of the developed scaffolds. Moreover, the mechanical properties should be investigated once the host tissues have infiltrated into the pores and the load-bearing functions are synergistically exerted by the integrated polymeric and biological phases [46]. In comparison with the scaffolds made of PHBHHx [34] or its blend with PCL [46], which have a similar layered fibrous structure and are fabricated by CAWS, the scaffolds obtained in this work showed a much higher compressive modulus, maybe as a consequence of the different morphological parameters (i.e., different pores size) and of the higher stiffness of PHBV in comparison with PHBHHx, as a consequence of its higher crystallinity [34].

The in vitro biological evaluation showed that the fabricated scaffolds supported the proliferation of a murine preosteoblastic cell line. The highlighted trend of differences in the cell viability among the different tested samples could be related to the differences in the various scaffold properties, such as the surface morphology, fiber diameter, pore size, and surface wettability. For instance, the higher surface roughness of the PP_10 scaffolds in comparison with the other scaffolds, as qualitatively shown by SEM analysis (Figure 3), could have played a key role in the limited cell viability observed after 2 h of cell culture for this kind of sample. As reported in the literature, a high surface roughness can determine a significant decrease in the surface wettability on a micro-/nanoscale level, negatively influencing the density and conformation of adsorbed binding proteins (e.g., fibronectin and vitronectin) onto the polymer matrix surface, as well as the strength of the protein/polymer interaction, thus limiting cell attachment and spreading [74]. The limited cell viability observed at day 7 for all samples could be related to the large scaffold pore size (>200 μm), which did not facilitate the retention of a significant number of cells during the seeding procedure, in agreement with the results obtained in this study for cell viability at 2 h as well as during previous relevant studies [35]. It should also be taken into account that these values are expressed as the percentage of the obtained absorbance value in comparison to that of the TCPS culture at the same time point, as well as that the samples were removed from the well before WST-1 analysis. As a consequence, only the cells that were effectively adhered to the polymer matrix and not those on the bottom of the well were quantified. This is another aspect that likely contributed to the low viability values obtained at day 7. In general, significantly higher cell proliferation was observed in the scaffolds composed of blends richer in PLGA. This result can be correlated to different scaffold features affected by the differences in the blend composition and scaffold geometrical properties. Usually, the tissue growth rate graph exhibits a typical S-shape characterized by a first lag stage, an intermediate range related to the steady-state tissue growth, and a final plateau resulting from the equilibrium between cell proliferation and the cell death rate [75]. Since the lag time is mainly dependent on the adhesion substrate material, it can be speculated that the observed differences in surface roughness and contact angle among the different samples could have affected the cell viability at the first time points. In addition, previous studies have demonstrated that the steady state tissue growth is significantly affected by a scaffold’s geometrical features, such as the curvature of the pores [75]. In particular, it was found that a higher surface curvature can determine a higher tissue growth rate [76], and, according to Fenchel’s theorem [77], fibers with a smaller radius have superior curvature. As a consequence, the higher cell proliferation rate of the PP_40 scaffolds at the first experimental time points can be at least partially explained by considering that they had the smallest fiber diameter and consequently the highest fiber curvature.

The significant increase in ALP values over the cell culture duration observed for scaffolds richer in PLGA is strictly related to the relevant cell proliferation trend, i.e., the enhancement of the cell viability value at a given experimental point by increasing the PLGA percentage in the blend. Indeed, the expression of high levels of ALP is favored by an adequate MC3T3-E1 cell confluence on the 3D construct [34]. This aspect can also, at least in part, explain the significantly higher levels of ALP activity observed at day 35 and 42 for some scaffolds richer in PLGA. The direct influence of the blend composition on ALP synthesis may be also possible, but further investigations are needed to clarify this aspect. Future studies on gene expression will contribute to clarifying whether the material composition itself significantly influences osteogenic synthesis.

Blending PHBV with PLGA has been previously investigated as an effective means of tuning the processing properties, hydrophilicity, and biodegradation rate of the resulting blend for different tissue engineering purposes. PHBV microsphere-embedded PLGA scaffolds produced by solvent casting/particulate leaching were investigated for their ability to support human mesenchymal stem cell proliferation [78]. Hepatocyte-growth-factor-encapsulated PHBV/PLGA microsphere scaffolds were demonstrated to be a suitable platform to maintain the viability and phenotype of primary hepatocytes [79]. PHBV/PLGA-blend nanofibrous scaffolds produced by electrospinning supported the proliferation of human skin fibroblast cells [80]. However, to the best of the authors’ knowledge, this is the first study describing the application of AM to PHBV/PLGA blends. A previous article by our research group [35] investigated CAWS-fabricated scaffolds made of another PHA (i.e., PHBHHx) for bone tissue regeneration. The described in vitro investigations were focused on the same cell line employed in this study for a shorter cell culture period (7 and 16 days), resulting in comparable values of cell viability.

## 4. Materials and Methods

### 4.1. Materials

Poly(3-hydroxybuthyrate-*co*-3-hydroxyvalerate) (PHBV) (hydoxybutyrate/hydroxyvalerate = 81/19 mol. %, Mw = 151.000 g/mol) was supplied by PHB Industrial S.A.—BIOCYCLE^®^ (Serrana, Brazil). Poly(D,L-lactide-*co*-glycolide) (D,L lactide/glycolide = 75/25 mol. %, Mw = 121.800 g/mol) was supplied by Vornia Biomaterials (Dublin Ireland). Chloroform (CHCl_3_) and ethanol (EtOH) were purchased from Carlo Erba (Milan, Italy) and used as received, without further purification.

### 4.2. Scaffold Fabrication

PHBV mixtures were prepared by suspending the polymer in CHCl_3_ (20% *w*/*v*) under magnetic stirring for 14 h at 35 °C and 34 h at room temperature. For the preparation of PHBV/PLGA suspensions, PHBV was suspended in CHCl_3_ under magnetic stirring for 7 h at 35 °C and 17 h at room temperature. The desired amount of PLGA was then added to the polymer suspension, and the obtained mixture was left under magnetic stirring for 7 h at 35 °C and 17 h at room temperature. Blends with a 20% *w*/*v* total concentration of the polymeric phase and different PHBV/PLGA weight ratios (i.e., 90:10, 80:20, 70:30, 60:40, and 50:50 wt. %) were prepared. Scaffolds were fabricated by means of a multifunctional AM machine with switchable operating heads developed by Fabrica Machinale S.r.l (Pisa, Italy). The machine is an industrialized prototype developed to process polymeric materials through melt- or solution-extrusion AM by changing the operating head, as depicted in Figure 1. This can be achieved through three spheres on the support plate holding the extrusion head that are connected to magnets with spherical bearing surfaces at the end of the 6 carbon tubes. The desired polymeric suspension was placed into a glass syringe fitted with a metallic needle (Gauge 22) and injected at a controlled feeding rate directly into an EtOH bath using the syringe pump system. Scaffold fabrication was carried out employing a deposition trajectory aimed at the production of scaffold with a 0–90 ° lay-down pattern, distance between fiber axis (d_x-y_) of 500 µm, and layer thickness (d_z_) of 100 µm. The optimized initial distance between the tip of the needle and the bottom of the coagulation bath (Z_0_) was 2 mm. The effect of different processing parameters, such as the solution feed rate (F), deposition velocity (V_transl_), and temperature of the coagulation bath (T_coag_), was evaluated to fabricate scaffolds made of PHBV or PHBV/PLGA blends with different ratios between the two copolymers (Table 1). Prism-shaped scaffolds with a theoretical square base of 12 mm length and a designed height of 1.5 or 5 mm were fabricated by employing optimized fabrication parameters. The 1.5 mm thick scaffolds were employed for SEM analysis and physicochemical, thermal, and biological characterization, while the 5 mm thick ones were employed for the porosity measurements and mechanical characterization. After fabrication, the scaffolds were removed from the coagulation bath, submitted to an optimized postprocessing treatment, and finally to vacuum drying for 7 h.

### 4.3. Film Preparation

Films made of PHBV or PLGA for contact angle measurements were prepared by solvent casting of a 2% *w*/*v* polymer/CHCl_3_. The solvent was added to the polymer in a conical flask, and the resulting mixture was left under magnetic stirring at 35 °C for 1 h. Polymeric mixtures were casted on square coverslips of 1.8 cm length and kept in a chamber saturated with solvent vapor for 8 h.

### 4.4. Morphological Characterization

Sample top-views and cross-sections, the latter obtained by fracture in liquid nitrogen, were analyzed by means of scanning electron microscopy (SEM, JEOL JSM 300, Tokyo, Japan). The average fiber diameter and pore size, defined as the distance between two adjacent fibers, were measured by Image J 1.43u software on top-view micrographs with 200× magnification. Data were calculated over 25 measurements per scaffold. Scaffolds’ overall porosity was evaluated according to the following equation:

(1) $$\mathrm{Porosity}\;\left(\%\right)=\left(\;\frac{V-V_{f}}{V}\right)\;\times \;100$$

where V is the total volume occupied by the structure, measured using a caliber, and V_f_ is the volume occupied by a scaffold’s fibers, measured by means of a liquid displacement method involving the immersion of the scaffold in a known volume of EtOH and the measurement of its increase, due to the volume occupied by the scaffold’s fibers. Five samples for each kind of scaffold were analyzed.

### 4.5. Viscosity Measurements

The viscosity of PHBV and PHBV/PLGA suspensions was measured using a viscometer PCE-RVI 2 V1R (PCE Instruments, Italy) on 50 mL of sample prepared as described above. The analysis was carried out at ambient temperature with a 3 cm diameter rotor on a single sample for each kind of polymeric suspension.

### 4.6. PLGA Extraction and ^1^HNMR Analysis

One representative scaffold (PP_20) was immersed in acetone at 25 °C for 48 h to selectively extract PLGA from the scaffold. After scaffold removal from the acetone bath, the liquid was evaporated under vacuum until a constant weight of the extracted polymer was obtained. The extracted polymer was then dissolved in deuterated chloroform and ^1^HNMR analysis of the extracts was carried out using a Varian Gemini 200 spectrometer (Varian Japan Co., Ltd., Tokyo, Japan) interfaced with a Sparc4 (Sun) console and VNMR6.1B software. The leached scaffold was vacuum dried for 7 h and its cross-section, obtained by fracture in liquid nitrogen, was observed by employing the SEM analysis procedure described above.

### 4.7. Contact Angle Measurements

Measurements of static water contact angle in air were carried out on the prepared polymeric scaffolds and films. Contact angle (ϴ) was determined by the sessile drop method using an FTA 200 Camtel goniometer (First Ten Ångstroms, Portsmouth, VA, USA). HPLC-grade water droplets were applied on each sample and ϴ was calculated. Ten samples for each kind of film/scaffold were characterized.

### 4.8. Thermal Characterization

Thermal properties of the scaffolds were evaluated by means of thermogravimetric analysis (TGA), standard differential scanning calorimetry (DSC), and modulated DSC (MDSC). TGA was performed using a TGA Q500 instrument (TA Instruments, Milan, Italy) in the temperature range of 30–700 °C, at a heating rate of 10 °C/min, and under a nitrogen flow of 60 mL/min. Scaffolds’ thermal degradation was evaluated by analyzing weight and derivative weight profiles as a function of temperature.

DSC analysis was performed using a Mettler DSC-822 instrument (Mettler Toledo, Milan, Italy). The samples were subjected to two heating cycles in the range of −20–200 °C at a heating rate of 10 °C/min, separated by a cooling cycle at a rate of −20 °C/min and by two isothermal steps, the first one at 200 °C and the second one at −20 °C. The analyses were carried out under a nitrogen flow of 80 mL/min. By considering the thermograms related to the first and the second heating cycles, polymer glass transition temperature (T_g_), melting temperature (T_m_), and enthalpy (ΔH_m_), cold crystallization temperature (T_cc_) and enthalpy (ΔH_cc_) were evaluated for each blend.

MDSC analysis was performed using a Discovery DSC 250 instrument (TA Instruments, New Castle, DE, USA) under a nitrogen flow of 330 mL/min, in the range of −20–200 °C, at a heating rate of 2 °C/min with a modulation period of 60 s, an amplitude of 0.32 °C, and a cooling rate of 20 °C/min. Three samples for each kind of polymer/scaffold were analyzed.

### 4.9. Mechanical Characterization

Scaffolds’ mechanical properties were analyzed under compression using an Instron 5564 uniaxial testing machine (Instron Corporation, Norwood, MA, USA) equipped with a 2 kN load cell. Scaffolds were tested either in dry conditions or after incubation in phosphate buffer saline (PBS) 0.01 M at 37 °C for 18 h. Five replicates were tested for each kind of scaffold and test. Samples were tested at a constant crosshead displacement of 0.5 mm/min between two parallel steel plates. The stress was defined as the measured force divided by the total area of the apparent cross-section of the scaffold, whilst the strain was evaluated as the ratio between the height variation and the initial height of the scaffold. Stress–strain curves were obtained from the software recording the data (Merlin, Series IX, Instron Corporation, Norwood, MA, USA). Compressive modulus (E) was calculated from the stress–strain curves as the slope of the initial linear region. The stress corresponding to 50% strain (σ_50_) and the linear elastic recovery (H_Rec_), defined as the percentage ratio between scaffolds’ height after testing and initial height, were also calculated. Five samples for each kind of scaffold were analyzed.

### 4.10. In Vitro Biological Characterization

#### 4.10.1. Cell Culture

Mouse-calvaria-derived preosteoblast cell line MC3T3-E1 was obtained from the American Type Culture Collection (ATCC CRL-2593, Manassas, VA, USA) and cultured in Alpha Minimum Essential Medium (α-MEM, Sigma, Milan, Italy), supplemented with 2 mM L-glutamine, 10% fetal bovine serum, 100 U/mL:100 µg/mL penicillin:streptomycin solution, and 5 µg/mL Plasmocin. Before experiments, cells were trypsinized with 600 µL trypsin–EDTA solution and resuspended in complete α-MEM at a concentration of 1 × 10^4^/mL for the evaluation of cell viability at 2 h, and at a concentration of 3 × 10^4^/mL for the evaluation of cell viability at the subsequent experimental time points (day 7 to 42). Scaffolds were seeded with 50 µL of cell suspension for the evaluation of cell viability at 2 h and with 300 µL for the evaluation of cell viability at the subsequent time points; the final volumes were adjusted, respectively, to 100 µL and 1 mL with complete medium. The specimens were then placed in an incubator with humidified atmosphere at 37 °C in 5% CO_2_. Osteogenic differentiation was induced 24 h after seeding by culturing cells in osteogenic medium prepared with α-MEM supplemented with γ-irradiated L-ascorbic acid (50 μg/mL) and β-glycerolphosphate (10 mM). The culture medium was replaced every 48 h and its orangey-red hue, due to supplementation with the indicator dye Phenol Red, was monitored in order to guarantee the correct physiological pH. Biological characterizations were carried out weekly at days 7, 14, 21, 28, 35, and 42; cells grown onto tissue culture polystyrene plates (TCPS) were used as control.

#### 4.10.2. Cell Viability and Proliferation

Cell viability was quantitively analyzed using the 4-[3-(4-iodophenyl)-2-(4-nitrophenyl)-2H-5-tetrazolium]-1,3-benzene disulfonate (WST-1) assay (Roche Molecular Biochemicals, Roche S.p.a., Monza, Italy), which is based on mitochondrial conversion of the tetrazolium salt WST-1 into soluble formazan in viable cells. In particular, cell viability was quantified at 2 h to assess the ability of the developed scaffolds to sustain cell colonization a short time after seeding, and then every 7 days up to 6 weeks of cell culture to evaluate cell proliferation. For analysis, the cells/scaffold construct was removed from the well and incubated in the WST-1 reagent diluted to 1:10 for 4 h at 37 °C and 5% CO_2_. Measurements of formazan dye absorbance were carried out with a Biorad microplate reader at 450 nm, with the reference wavelength at 655 nm. Cell viability was expressed as percentage absorbance in comparison to the value obtained for the control (TCPS) at the same time point. The in vitro cell viability test was performed on three samples for each kind of scaffold.

#### 4.10.3. Morphological Characterization by Confocal Scanning Laser Microscopy (CLSM)

Cells were fixed with 3.8% *w*/*v* paraformaldehyde in PBS 0.01 M pH 7.4, permeabilized with a PBS 0.01 M/Triton X-100 solution (0.2% *w*/*v*) for 10 min, then treated with a PBS 0.01 M/bovine albumin serum (0.1% *w*/*v*) for 30 min, and finally incubated at room temperature in the dark with a PBS 0.01 M solution of Alexa Fluor 488 phalloidin (Invitrogen^TM^, Thermo Fisher Scientific, Monza, Italy) and 4′-6-diamidino-2-phenylindole (DAPI; Invitrogen^TM^, Thermo Fisher Scientific, Monza, Italy) for 60 and 30 min, respectively. After dye incubation, samples were washed with PBS 0.01 M before being mounted on a glass slide and sealed with a resin for microscopic observation. A Nikon Eclipse TE2000 inverted microscope equipped with an EZ-C1 confocal laser and a Differential Interference Contrast (DIC) apparatus was used to analyze the samples (Nikon, Tokyo, Japan). A laser diode (405 nm emission) and an argon ion laser (488 nm emission) were used to excite DAPI and Alexa fluorophores, respectively. Images were captured with Nikon EZ-C1 software with identical settings for each sample. Images were further processed with GIMP (GNU Free Software Foundation) Image Manipulation Software and merged with Nikon ACT-2U software.

#### 4.10.4. Cell Differentiation

The differentiation of MC3T3-E1 cells towards the osteoblastic phenotype was evaluated by measuring the alkaline phosphatase activity (ALP), using a colorimetric method after 7, 14, 21, 28, 35, and 42 days of cell culturing. The test is based on the conversion of p-nitrophenyl phosphate into p-nitrophenol in the presence of alkaline phosphatase. The seeded scaffolds were treated as previously reported. Briefly, scaffolds were washed with DPBS and then placed in 1 mL lysis buffer, pH 10. The scaffolds were then submitted to three freeze–thaw cycles for at least 1 h for each cycle [43]. Following this treatment, the supernatants were taken from the samples and added to p-nitrophenyl phosphate substrate (Sigma, Milan, Italy). A standard calibration was prepared using alkaline phosphatase from bovine kidney (Sigma, Milan, Italy). The reaction was performed at 37 °C for 30 min and the absorbance was read at 405 nm by using Benchmark microplate reader (Bio-rad) (Hercules, CA, USA). The results of ALP assay were normalized with the total protein content of each sample, which was measured using a Pierce^TM^ micro-BCA protein assay (Thermo Fisher Scientific, Waltham, MA, USA), and were reported as nM substrate converted into product/min and /mg protein. ALP analysis was carried out on three samples for each kind of scaffold.

### 4.11. Statistical Analysis

The data are represented as mean ± standard deviation. Statistical differences were analyzed using one-way or two-way (when indicated) analysis of variance (ANOVA), and a Tukey test was used for post hoc analysis. A *p*-value < 0.05 was considered statistically different.

## 5. Conclusions

This study has shown for the first time that CAWS can be employed to fabricate PHBV-based scaffolds with a predefined shape, size, and 3D porous architecture. In particular, blending PHBV with another biodegradable polyester, i.e., PLGA, was shown to be a valuable and easy way to control and enhance the scaffold’s morphological features, wettability, mechanical parameters, cytocompatibility, and bioactivity. Blending two immiscible polymers resulted in the scaffold’s fibers being composed of two different polymeric phases: a PHBV-based phase forming a continuous matrix in which a PLGA-based phase was dispersed. The morphology of the dispersed phase varied with the blend composition; in particular, it assumed the shape of microfibrils oriented along the longitudinal fiber axis in the case of the PP_20 scaffolds, as a consequence of shear forces acting on the polymeric mixture during extrusion. This kind of morphology could be particularly interesting for the incorporation of drugs into a scaffold’s polymeric matrix. Indeed, the developed scaffolds could be studied for the selective loading of a bioactive agent in only one phase, as well as the loading of different drugs in the two phases by exploiting the immiscibility of PHBV and PLGA and/or their (in)solubility in different solvents. Cutting-edge advancements in precise and sustained drug-release profiles or the sequential delivery of multiple drugs from the same scaffold can be based on this biphasic scaffold approach [81,82]. Further studies will be carried out to determine the kinetics of in vitro degradation of the developed polymeric blends as well as the rate of bioerosion of the resulting polymeric scaffolds. Furthermore, Atomic Force Microscopy analysis will be carried out on the fabricated scaffolds to measure the surface roughness, since it is a key parameter influencing cell proliferation and differentiation.

## Figures and Tables

**Table 1:** Optimized CAWS processing parameters for scaffolds with different PHBV/PLGA ratios.

Scaffold	PHBV/PLGA [*w*/*w*%]	F [mL/h]	V_transl_ [mm/min]	T_coag_ [°C]	Post-Processing
PP_0	100:0	2.1	1200	38–39	- EtOH immersion (6 days) - Kept under a fume hood (48 h) - Vacuum drying at RT (6 h)
PP_10	90:10	2.3	1200	41–42	
PP_20	80:20	2.3	1200	42–43	- Pressure applied on scaffold’s top surface during drying under a fume hood (48 h) - Vacuum drying at RT (6 h)
PP_30	70:30	2.3	1200	42–43	
PP_40	60:40	2.3	1200	43–44	
PP_50	50:50	2.3	600	46–47	

F: extrusion flow rate; V_transl_: needle translation velocity; T_coag_: coagulation bath temperature; RT: room temperature.

**Table 2:** Data relevant to the structural parameters of the scaffolds.

Sample	Fiber Diameter (µm)	Pore Size (µm)	Porosity (%)
PP_0	112 ± 8	268 ± 34	75 ± 14
PP_10	110 ± 12	249 ± 40	73 ± 10
PP_20	110 ± 13	237 ± 37 *	70 ± 8
PP_30	130 ± 22 ^a^	296 ± 72 *^,^**	68 ± 10
PP_40	88 ± 9 ^a^	360 ± 96 ^a^	70 ± 12
PP_50	150 ± 13 ^a^	227 ± 64 **	70 ± 13

All data are reported as average ± standard deviation (25 measurements carried out on 1 micrograph for each kind of scaffold). *,** For a given parameter, pairs of values marked with the same number of asterisks were statistically different (*p* < 0.05). ^a^ For a given parameter, marked data are statistically different from all the others (*p* < 0.05).

**Table 3:** Data relevant to TGA characterization.

Sample	DTG	
	T_max1_ (°C)	T_max2_ (°C)
Raw PHBV	244 ± 6 *	-
Raw PLGA	-	309 ± 8 ^a^
PP_0	271 ± 4	-
PP_10	236 ± 3 *	-
PP_20	276 ± 5	348 ± 8
PP_30	273 ± 4	334 ± 15
PP_40	274 ± 6	338 ± 4
PP_50	283 ± 4	337 ± 7

All data are expressed as average ± standard deviation (measurements carried out on 3 samples for each kind of scaffold). * Data marked with this symbol are statistically different from those without the symbol, but not between themselves (*p* < 0.05). ^a^ Statistically different from all other values (*p* < 0.05).

**Table 4:** Data relevant to the thermal characterization by DSC analysis.

Sample	First Heating	Second Heating	Sample	First Heating	Second Heating	Sample	First Heating	Second Heating
	T_g_ (°C)	T_m_ (°C)		T_g_ (°C)	T_m_ (°C)		T_g_ (°C)	T_m_ (°C)
Raw PHBV	-	164.0 ± 1.0	76.0 ± 4.4	2.4 ± 0.1 ^a^	58.0 ± 1.0	43.8 ± 1.0 *	163.0 ± 1.0	64.0 ± 1.0 *
Raw PLGA	50.3 ± 0.4	-	-	50.1 ± 0.2	-	-	-	-
PP_0	-	165.4 ± 0.5	78.9 ± 0.1 ^a^	3.2 ± 0.7	64.6 ± 2.0	44.7 ± 1.0 *	164.5 ± 1.0	63.9 ± 0.6 *
PP_10	59.3 ± 0.4	164.6 ± 0.7	58.4 ± 3.1	3.8 ± 0.2	57.0 ± 3.0	30.9 ± 0.6	163.9 ± 0.4	49.0 ± 0.3
PP_20	59.3 ± 0.1	163.6 ± 0.3	57.8 ± 1.5	3.6 ± 0.1	59.0 ± 2.0	31.5 ± 0.3	163.5 ± 0.2	48.3 ± 1.0
PP_30	59.0 ± 0.2	165.1 ± 0.7	60.9 ± 3.0	3.6 ± 0.3	56.0 ± 1.0	30.1 ± 2.3	164.1 ± 0.4	52.9 ± 1.0
PP_40	59.5 ± 0.1	163.9 ± 0.3	45.2 ± 0.7	3.6 ± 0.1	61.2 ± 0.4	21.7 ± 0.4 ^a^	163.7 ± 0.2	35.5 ± 2.0 ^a^
PP_50	58.0 ± 3.0	163.2 ± 0.1	38.0 ± 0.7	3.1 ± 0.3	61.0 ± 1.0	16.9 ± 0.1 ^a^	163.3 ± 0.3	27.8 ± 0.3 ^a^

All data are expressed as average ± standard deviation (measurements carried out on 3 samples for each kind of sample). * Data marked with this symbol are statistically different from those without the symbol, but not between themselves (*p* < 0.05). ^a^ Marked data are statistically different from all the others (*p* < 0.05).

**Table 5:** Compressive mechanical parameters of scaffolds tested in dry or wet (PBS 0.01 M, 37 °C) conditions.

Sample	Dry			Wet		
	E (MPa)	σ_50_ (MPa)	H_Rec_ (%)	E (MPa)	σ_50_ (MPa)	H_Rec_ (%)
PP_0	1.1 ± 0.4	0.4 ± 0.1	30.4 ± 3.9	1.0 ± 0.3	0.3 ± 0.2	28.2 ± 4.0
PP_10	1.0 ± 0.4 *	0.4 ± 0.1	32.7 ± 8.6	2.1 ± 0.6 *	0.4 ± 0.1	35.6 ± 4.6
PP_20	1.4 ± 0.3	0.5 ± 0.2	36.2 ± 10.9	1.5 ± 0.3	0.4 ± 0.1	30.1 ± 5.1
PP_30	1.0 ± 0.4	0.5 ± 0.2	34.4 ± 8.3	1.7 ± 0.8	0.6 ± 0.3	33.9 ± 9.5
PP_40	1.1 ± 0.4	0.7 ± 0.4	31.0 ± 3.0	1.3 ± 0.4	0.5 ± 0.1	38.2 ± 9.8
PP_50	3.4 ± 2.1 ^a^	2.2 ± 1.9	36.1 ± 9.2	3.7 ± 1.1 ^a^	1.8 ± 1.0	51.0 ± 17.0

Data are reported as average ± standard deviation (measurements carried out on 5 samples for each kind of scaffold). ^a^ Data marked with the same symbol are statistically different from all the others relevant to E, but not between themselves (*p* < 0.05). * Data marked with the same symbol are statistically different (*p* < 0.05).

## Data Availability

Not applicable.

## Supplementary Materials

The following supporting information can be downloaded at: https://www.mdpi.com/article/10.3390/ijms23073895/s1.
