# Supplementary material for: Additive Manufacturing of Poly(3-hydroxybutyrate-co-3-hydroxyvalerate)/Poly(D,L-lactide-co-glycolide) Biphasic Scaffolds for Bone Tissue Regeneration
Source: Int J Mol Sci. 2022 Mar 31;23(7):3895. doi: 10.3390/ijms23073895 (PMC8999344; doi:10.3390/ijms23073895)
Supplement: Supplementary file 1 [file ijms-23-03895-s001.zip › ijms-1635436-supplementary/ijms-1635436-supplementary.pdf]

Supplementary materials

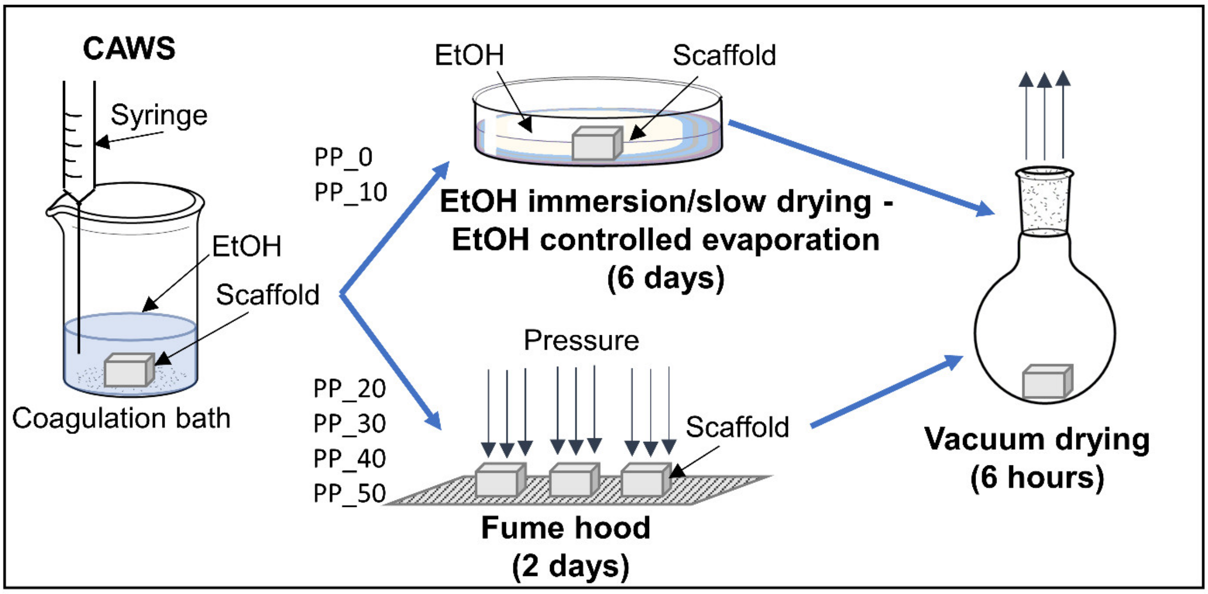

Figure S1 Schematic representation of the post-processing steps carried out on the fabricated scaffolds
